# Oscillations emerging from noise-driven steady state in networks with electrical synapses and subthreshold resonance

**DOI:** 10.1038/ncomms6512

**Published:** 2014-11-18

**Authors:** Tatjana Tchumatchenko, Claudia Clopath

**Affiliations:** 1 Department Theory of Neural Dynamics, Max Planck Institute for Brain Research, Max-von-Laue Strasse 4, 60438 Frankfurt am Main, Germany; 2 Department of Bioengineering, Imperial College London, South Kensington Campus, London SW7 2AZ, UK

## Abstract

Oscillations play a critical role in cognitive phenomena and have been observed in many brain regions. Experimental evidence indicates that classes of neurons exhibit properties that could promote oscillations, such as subthreshold resonance and electrical gap junctions. Typically, these two properties are studied separately but it is not clear which is the dominant determinant of global network rhythms. Our aim is to provide an analytical understanding of how these two effects destabilize the fluctuation-driven state, in which neurons fire irregularly, and lead to an emergence of global synchronous oscillations. Here we show how the oscillation frequency is shaped by single neuron resonance, electrical and chemical synapses.The presence of both gap junctions and subthreshold resonance are necessary for the emergence of oscillations. Our results are in agreement with several experimental observations such as network responses to oscillatory inputs and offer a much-needed conceptual link connecting a collection of disparate effects observed in networks.

## Abstract (web summary)

Oscillatory activity is ubiquitous in the brain and plays a critical role in cognitive processing. Tchumatchenko and Clopath present a model whereby oscillatory activity emerges through subthreshold resonance and electrical coupling between inhibitory interneurons.

## Graphical abstract

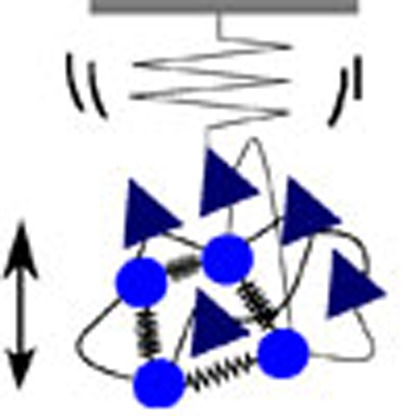

Cognitive phenomena such as conscious perception and attention are associated with cortical oscillatory activity in the frequency range from lower than one to a few hundred Hertz^1^,^2^,^3^,^4^. A number of theories have been proposed for the generation of oscillatory rhythms that emphasize the importance of a specific subtype of neurons and their synaptic connections. Inhibitory interneurons are implicated in oscillatory cortical activity in many brain regions. An important feature of inhibitory interneurons is the presence of a subthreshold resonance that selectively amplifies firing rate response to select frequencies already present in isolated neurons^5^,^6^,^7^,^8^,^9^. This alone could suffice for the inhibitory interneurons to be the driving force behind global network oscillations. In addition, those same inhibitory interneurons are coupled by gap junctions in many cortical regions where global network oscillations are reported^10^,^11^,^12^,^13^. It is therefore conceivable that gap-junction-induced synchrony and subthreshold oscillations provide a substrate for global oscillations in any regions where they co-occur. Although a large number of theoretical studies have tackled the effects of chemical synapses on synchrony and global oscillations, the role of electrical synapses has received less attention and has been mostly studied in networks with identical neurons in the low noise limit and largely without considering the frequency preferences of single neurons^13^,^14^,^15^,^16^,^17^. In physiological conditions *in vivo*, neurons receive noisy input and fire irregularly a situation where global oscillations could be difficult to achieve and the synchronizing effect of gap junctions and single neurons could be cancelled out. It is therefore important to understand how the single neural properties interact with chemical and electrical connectivity in the noisy fluctuation-driven regime, in which neurons fire irregularly because they are driven by noisy inputs, and how these networks can transition to global oscillations and synchrony. The current lack of theoretical approaches so far is largely due to the technical challenges of solving multiple coupled Fokker Planck equations that are necessary for the inclusion of gap junctions and single-neuron frequency preference. Therefore, most of the computational studies consider either gap junctions or resonant single neurons, never both simultaneously and largely focus on numerical simulations^18^,^19^,^20^,^21^,^22^.

To overcome these difficulties and address analytically the interplay between the single neuron biophysics and the chemical and electrical connectivity, we develop an adaptive threshold model framework, where we consider neurons in the fluctuation-driven regime, that is driven by largely fluctuating inputs, that results in irregular firing, while neglecting their reset. Inhibitory neurons have a subthreshold resonance, they are connected electrically among themselves and chemically to excitatory neurons. Excitatory neurons do not exhibit subthreshold resonance and are chemically coupled. Within this framework we compute the sub- and superthreshold resonant properties of single neurons and use this framework to set up a self-consistent mean-field description. Using the mean-field approach, we compute the network dynamics in response to an incoming oscillatory current. Notably, we show that the resonance frequency of the network reflects directly the subthreshold preference in individual neurons of a subset of neurons, while chemical synapses act as a multiplicative gain that regulates the amplitude of this response but not its shape.

We then derive the oscillatory instability condition for the network to transition from irregular spiking to global self-sustained oscillations. In the oscillatory state, the neurons fire synchronously and therefore are no longer in the fluctuation-driven regime. We demonstrated analytically two requirements for the emergence of self-sustained global oscillations from the fluctuation-driven state, (i) frequency preference in the inhibitory neurons and (ii) inhibitory neurons connected with gap junctions, where the gap-junction-induced spikelet is larger than the γ-aminobutyric acid (GABAergic) inhibitory postsynaptic current (IPSC), and leads to global excitatory coupling. It is important to emphasize that in our model synchronous global oscillations are characterized by one spike per neuron and cycle, and emerge from the irregular fluctuation-driven network state. The frequency of global oscillations is largely defined by single neuron subthreshold oscillations. Notably, this is a fundamentally different mechanism compared with previous studies that describe the emergence of sparsely synchronized fast oscillations^23^, or gap-junction-mediated synchronous oscillations in networks without subthreshold frequency preference^24^ or global oscillations in network-coupled oscillators^15^.

Here we demonstrate that the theoretical predictions we derived are consistent with the recent physiological evidence from cell-specific light stimulation in the barrel cortex^22^. Adapting our framework to the cell properties of the barrel cortex, we show that the excitatory neurons amplify low frequencies at the network level, whereas the inhibitory neurons alone can support a resonance in the γ-range, which is consistent with ref. 22. Our analytical adaptive framework is not limited to the case of the barrel cortex, but can be applied to any neuronal network in the fluctuation-driven state with subthreshold resonance and gap junctions.

## Results

### Subthreshold resonance leads to firing rate resonance

**Figure 1:**
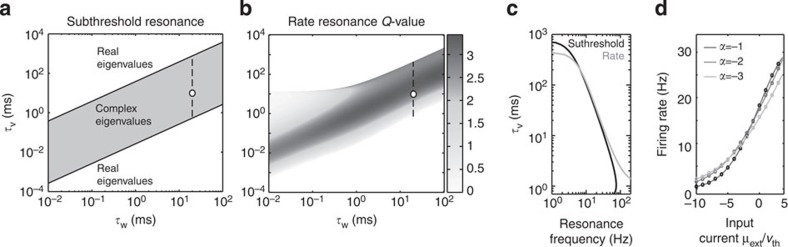
Subthreshold and firing rate resonances of single neurons and the ensuing firing activity. (**a**) Subthreshold phase-plane in single neurons; subthreshold oscillations can be observed for complex eigenvalues of subthreshold transformation in equation (1). (**b**) *Q*-value of firing rate resonance in single neurons in the *τ*_V_, *τ*_w_-phase plane (dashed line in **a**); sharpest resonances (high *Q*) values are observed for parameter values corresponding to subthreshold oscillations in **a**. (**c**) Resonant frequencies for firing rate (grey) and subthreshold dynamics (black) at *τ*_w_=4.18 ms. (**d**) Firing rate of single neurons as a function of input current; solid line (theory) and dots (simulations). Parameters as in Table 1.

We model inhibitory neurons via a leaky voltage dynamics coupled with an adaptive variable. This model exhibits subthreshold oscillations that depend on the relative time constants of the voltage (*τ*_*V*_) and of the adaptive variable (*τ*_*w*_), as shown in Fig. 1a and mathematically derived in the Methods section. Next, we show the effect of this subthreshold resonance on the firing rate susceptibility to periodic inputs. Figure 1b demonstrates that the *Q*-value (peak/width) of the resonance peak is highest in the parameter regime where subthreshold resonance is most salient. The preferred frequency for the rate response is very closely related to the preferred subthreshold resonance frequency (Fig. 1b,c). Albeit sizable differences between rate and subthreshold resonance can be observed in Fig. 1c close to the edge of resonant parameter space. Importantly, this tight correspondence between subthreshold and rate resonance we observe for the threshold model framework is consistent with the previous observation by Richardson *et al.*^21^ They found that fluctuation-driven but not mean activity can unmask the subthreshold oscillations in the spike firing patterns of leaky integrator neurons. Finally, the predicted steady-state firing rate we calculated analytically is consistent with numerical simulations, Fig. 1d. The firing rate increases with the current drive and decreases with the adaptive coupling *α*, consistent with previous experimental and theoretical reports^21^.

### Response of a single neuron to an oscillatory current

**Figure 2:**
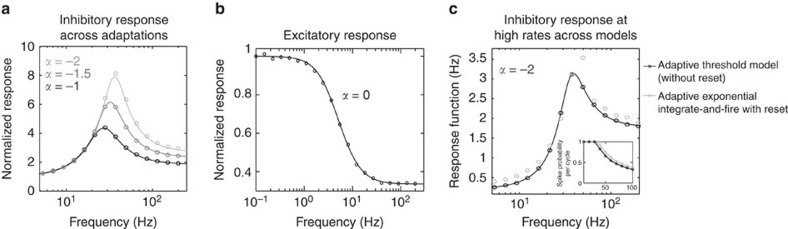
Inhibitory and excitatory linear firing rate response. Normalized linear rate response amplitude *R*(2*πf*)/*R*(0) as a function of input frequency. (**a**) Rate response exhibits a resonance in a population of inhibitory neurons with the threshold model at low-firing rate. The solid lines are the theory and the dots are the simulations for different values of *α*. (**b**) Rate response in the excitatory neurons exhibits a low-pass behaviour. (**c**) Comparison of the rate responses for the theory and different neuron models, where inhibitory neurons fire at high rate (black line: theory, black dots: adaptive threshold neuron, grey dots: aEIF model). Parameters as in Table 1.

To address how networks respond to dynamical stimuli, the first step is to understand how the firing rate of single neurons responds to weak sinusoidal current drive of frequency *f* (refs 19, 21). We therefore derive the linear response functions *R*_*j*_(*ω*) of a neuron receiving an oscillatory current (see Methods). Figure 2 demonstrates the linear response functions for inhibitory and excitatory neurons.

The salient feature of the inhibitory linear response is the resonance emerging from the adaptive voltage coupling *α*. Figure 2a shows that increases in *α* result in a higher resonance peak and a modest shift to higher frequencies. Figure 1b,c also demonstrate the firing rate frequency response and its sharpness in the frequency domain. *Q*-value that we use to quantify sharpness is defined as Δ*f*/*f*_*R*_ where Δ*f* is the full width at half maximum and *f*_*R*_ is the peak frequency. The excitatory neurons, however, show amplification of lower frequencies only Fig. 2b because, by assumption, they lack subthreshold adaption (*α*=0). Their firing rate response drops at higher frequencies due to the membrane time constant of the leaky integrator.

To show the robustness of our findings across different spiking models, we compare the threshold model with the adaptive exponential integrate-and-fire (aEIF) model, which is shown to reproduce different firing patterns^25^. In Fig. 2c, we show that the inhibitory response function in both models are very similar even at high rates, firing rate around 35 Hz in both models. A more detailed response functions comparison across models can be found in Figs 1 and 2 in the Supplementary Methods.

### Network response - Link to experiments

**Figure 3:**
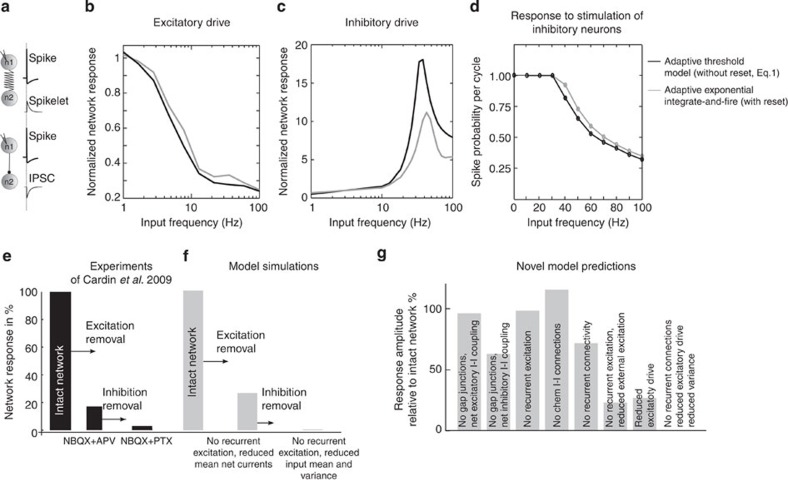
Network response amplitude in experiment and theory. (**a**) Spike-triggered effects in inhibitory neurons. Postsynaptic spikelet originating from gap-junction coupling (top) and the GABAergic-mediated IPSC (bottom). (**b**) Simulated network response *R*_E_(2*πf*)/*R*_E_(0) (normalized) for excitatory and (**c**) *R*_I_(2*πf*)/*R*_I_(0) inhibitory stimulation as a function of input frequency *f*. Parameters are in Table 1 (black: threshold model, grey: aEIF model). (**d**) Spike per cycle as a function of stimulation frequency (black: threshold model, grey: aEIF model). (**e**) Experimentally measured network local field potential (LFP) amplitude in response to light activation of inhibitory cells at 40 Hz (values adapted from Supplementary Fig. 9 in ref. 22) in two different network connectivity conditions (after excitation- and subsequent inhibition blockade) relative to the intact network. (**f**) Simulated network response of aEIF neurons at 40 Hz in the conditions similar to the experimental data (see Table 1). (**g**) Simulated network response of aEIF neurons at 40 Hz for different settings of gap-junction strength, recurrent connectivity, mean and variance of the drive.

Studying the network response to external periodic drive in Fig. 3, we discovered that stimulation of excitatory and inhibitory neurons yield qualitatively different results. If the oscillatory current is delivered to excitatory neurons, the network only amplifies lower frequencies (Fig. 3b). If the oscillatory current is delivered to inhibitory neurons, the network shows a resonance peak that resembles the rate resonance of single inhibitory neurons (Fig. 3c). This indicates that the location and width of the inhibitory subthreshold resonance rather than recurrent chemical synaptic connectivity is a major determinant of network dynamics in response to external stimuli. We note that the form of rate response functions does not depend on the details of the spike generation mechanism such as voltage reset and is preserved for the adaptive exponential and leaky integrate-and-fire models (see Fig. 3c the black and grey line, as well as Supplementary Fig. 1b). We therefore expect these results to generalize to other classes of networks where subpopulations of neurons exhibit differences in subthreshold frequency preferences.

Next, we explore whether parameter regimes exist where stimulation of the excitatory neurons could show signatures of the inhibitory subthreshold resonance. To investigate this hypothesis, we increased threefold the E to I connectivity strength Γ_IE_ and shortened the voltage time constant of the excitatory population *τ*_*V*,E_ to enable excitatory response function to cover the *γ*-frequency range. Supplementary Fig. 3 shows that these two interventions are sufficient to induce γ-band resonance on excitatory stimulation. However, it is hard to find experimental evidence supporting Γ_IE_>>Γ_EI_, Γ_EE_ such that we consider the parameter set of similar recurrent connectivities in Fig. 3 to be a more plausible case in cortical networks.

We find that results in Fig. 3 are consistent with the experimental finding of Cardin *et al.*^22^ in the barrel cortex *in vivo*. These experiments show that inhibitory neurons rather than excitatory neurons amplify *γ*-range frequencies. The external stimulation at varying stimulation frequencies in Cardin *et al.*^22^ corresponds to the network linear response we have studied in the previous two sections. In Fig. 3b–d, we show that our model can reproduce the high spike probability per cycle (around 0.9 for 40 Hz) and recover the frequency response dynamics found in neural networks under excitatory and inhibitory stimulation in Cardin *et al.*^22^

This framework offers us the opportunity to investigate the contribution of the single neuron properties and connectivity to the network response dynamics. Cardin *et al.*^22^ addressed this question by blocking successively excitatory and inhibitory connections. They showed that stimulating the inhibitory neurons optogenetically at 40 Hz lead to a large local field potential (LFP) response, which decreased by ~70% when the excitatory connections are blocked, and decreased to almost baseline when all the chemical connections were blocked (see Fig. 3e, figure redrawn with data from ref. 22). In Fig. 3f, we mimic this experiment and reproduce quantitatively the experimental results. However, in our model, blockade of connectivity is a combination of removing recurrent excitatory synapses and reducing external excitatory drive. Similarly, blockage of GABAergic connections correspond to a removing of inhibitory synapses in our model, increasing the network drive and reducing the variance of the noise. This is due to the fact that in the irregular activity of cortical neurons, the mean and the variance of the firing rate co-vary^26^.

Notably, other models such as PING/ING models have been tried to explain the findings of Cardin *et al.*^22^. However, Tiesinga and Sejnowski^27^ have shown that these networks fail in reproducing two major features of Cardin *et al.* results: (1) stimulation of excitatory neurons leads to *γ*-frequencies amplification unlike in Cardin *et al.* and (2) asynchronous and low-rate background activity is absent in PING/ING networks but is the defining feature of cortical networks^22^,^28^. Our own simulations of a PING/ING network where excitatory neurons are externally stimulated (Supplementary Fig. 4) also confirm Tiesinga *et al.* results and suggest that this model class is inconsistent with results by Cardin *et al.*

### Predictions for network’s response to an oscillatory drive

The response of a network subject to external stimuli is influenced by a number of effects such as external drive and recurrent chemical and electrical coupling. To consider each effect separately we turn to Fig. 3g. In Fig. 3g, we show the separate contribution of the mean drive, variance drive, recurrent connectivity and gap junctions on the network response to an oscillatory current at 40 Hz. We find that the inhibitory subthreshold resonance rather than recurrent chemical synaptic connectivity is a major determinant of network dynamics in response to external stimuli. We find the largest drop in response amplitude when excitatory external drive’s mean or variance is reduced. On the other hand, reductions in recurrent conductivities by and large introduce only modest effects. This could be tested experimentally by opto-genetically silencing the thalamic neurons providing inputs to the cortical neurons. This manipulation should show a drastic lowering of LFP γ-power. However, silencing the local cortical excitatory neurons should only show a subtle drop in *γ*-power. In summary, our findings support the hypothesis that intrinsic biophysics of excitatory cells is the primary cause for the reduction of excitatory neurons ability to respond primarily to low-frequency stimulation rather than synaptic recurrent connectivity, or even the recruitment of a distinct subgroup of low-threshold spiking neurons that selectively enhance lower-frequency oscillations^20^. Therefore, our study offers a complementary explanation for the observed experimental findings and supplements previous multi-compartment large-scale simulations by offering a compact analytical treatment that combines both electrical, chemical and single-neuron subthreshold contributions.

### Predictions for self-sustained network oscillations

In this section, we characterize the emergence of self-sustained global oscillations occurring when the asynchronous irregular network state becomes unstable with respect to spontaneous oscillatory perturbations. We consider the stability conditions outlined in refs 23, 29 and apply them to our network. Starting with an infinitesimal time-dependent perturbation in input current, we obtain changes firing rate via *R*_*j*_(*ω*) (equation (3)) and propagate these changes sequentially back to the current level via *S*_*j*_(*ω*) (equation (4)). The stability of this transformation is given in equation (6) and its two population analogon can be found in equation (47) in the Supplementary Methods. It is important to emphasize that these stability conditions can predict where this oscillatory transition boundary should occur in parameter space and what oscillatory frequency emerges at this transition.

**Figure 4:**
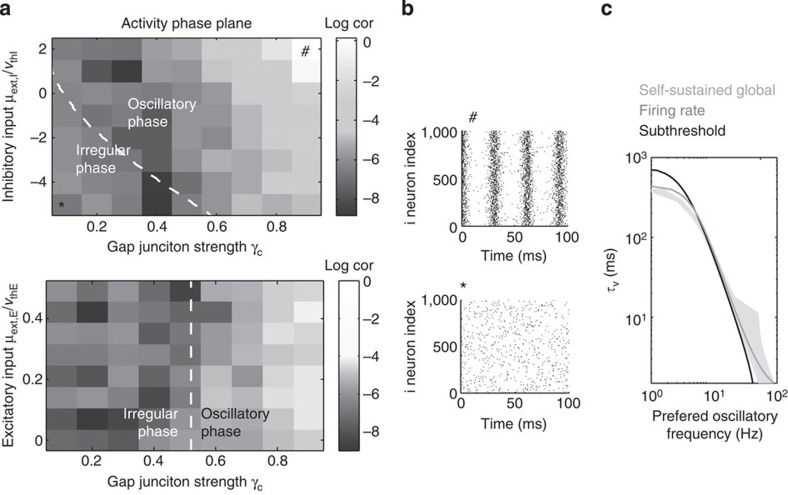
Global self-sustained oscillations and their frequency. (**a**) Phase transition to a global self-sustained oscillatory state as a function of gap-junction strength and current input to inhibitory neurons (top) and to excitatory neurons (bottom). Dashed white line indicates the phase transition between irregular and oscillatory state. Grey scale indicates the log network coherence computed numerically (see Methods, parameters in Table 1). Current input to excitatory neurons is less effective in eliciting global oscillations. (**b**) Spike rasters of the inhibitory neurons in the oscillatory (top, parameter choice indicated by # in **a**) and in the asynchronous irregular regime (bottom, parameter choice indicated by * in **a**). (**c**) Membrane constant as a function of oscillation frequency: for self-sustained global oscillation (grey area), firing rate resonance (dark grey) and subthreshold resonance (black). In all three conditions, oscillatory frequencies are closely related.

We also address the stability of the irregular steady state in numerical simulations and contrast these predictions with the above theory. Figure 4a,b illustrates the global network coherence (Cor, as defined in section ‘Quantification of global network coherence’) for a network as a function of gap-junction strength *γ*_c_ and normalized current drive *μ*_ext,I_/*V*_thI_. The dotted line shows the theoretical boundary, matching the very onset of global coherence computed numerically. We find good correspondence between the theoretically predicted stability boundary and numerical simulations, both for the threshold based model in Fig. 4a and the corresponding leaky and integrate-and-fire (EIF) models with reset (Supplementary Figs 1 and 2). As individual single-neuron response functions and network firing rates are not affected by the presence of voltage reset (see Supplementary Fig. 1b–d), we expect the oscillatory boundary derived here for the reset-free model to correspond to that of leaky integrate-and-fire and models with reset. Indeed, we find that the transition boundary to the global oscillatory state remains unchanged for the adaptive integrate-and-fire model as well as for the aEIF model^30^, which is known to reproduce different neuronal firing patterns^31^,^32^.

Figure 4b shows the corresponding spike rasters in the asynchronous irregular and in the oscillatory state. Note that in the oscillatory state, the neurons fire synchronously and therefore are not in the fluctuation-driven regime anymore. Increasing the excitatory drive *μ*_ext,E_/*V*_thE_ however does not lead to self-sustained oscillations (Fig. 4a, bottom), as shown numerically and theoretically. This is due to the assumption that excitatory neurons do not have a subthreshold resonance (*α*=0). In summary, we observe that inhibitory current is a potent drive of global self-sustained oscillations, while excitatory drive is less effective. In Fig. 4c, we show that the frequency of these global self-sustained oscillations corresponds closely to the subthreshold resonance as well as the rate preference (Fig. 1) of inhibitory neurons. The grey region in Fig. 4c delimits the frequency range of emerging self-sustained oscillations. Importantly, we show mathematically that the self-sustained oscillations can occur only if (1) the net effect of the GABAergic synaptic current and gap-junction-mediated spikelet is positive (Γ_II_>0) and (2) at least one subpopulation of the neurons expresses subthreshold oscillations (see Methods). We would like to emphasize that these requirements hold for a network transitioning from the fluctuation-driven regime. Of course, oscillations can occur through different mechanisms, such as through coupling of mean-driven neurons that effectively act as oscillators^1^,^13^,^14^,^15^,^33^, or through synaptic delays^23^,^29^.

## Discussion

We presented a comprehensive network study of the effects arising from a combination of subthreshold resonance, electrical and chemical synaptic coupling. In our analysis we focused on the fluctuation-driven network state expressed in many cortical regions^26^,^28^,^34^. Our results demonstrate that in a situation where neuronal networks are subject to external oscillatory stimuli, the subthreshold frequency preference of a subpopulation defines the global network frequency preference, while electrical and chemical connectivity provide multiplicative gain factors. When an external stimulus of varying frequency impinges on a subpopulation, not only the response amplitude of the stimulated population but also the complete network is closely related to the subthreshold frequency preference of the stimulated population. We found that the electrical synapses are effective at synchronizing the neuronal network if the spike triggered coupling, composed of the GABAergic current and the gap-junction-mediated current, is net positive and a subthreshold resonance is present in at least one subpopulation. The absence of a subthreshold resonance (*α*=0) stabilizes the irregular steady state and precludes the generation of a sustained global oscillation, regardless of electrical or chemical coupling. Notably, we find that subthreshold resonant frequency closely corresponds not only to the firing rate preference in independent neurons but also determines the frequencies of the global network oscillations emerging at the instability boundary of the irregular steady state.

Electrical synapses as well as subthreshold oscillations have been previously recognized as key ingredients in the generation of global rhythms^1^,^10^. Yet, typically, these two properties are studied separately in model settings. Here we briefly recapitulate previous results on the role of electrical synapses as well as subthreshold frequency preference and highlight their relation to our results and model. We start by considering the studies with a focus on neurons with subthreshold frequency preference and those describing the irregular steady state of neural networks. The irregular asynchronous steady state of cortical networks and its oscillatory instability has first been quantified by Brunel and Hakim^23^,^29^, and Brunel and Wang^35^ in a series of landmark papers. Using the Fokker-Planck formalism, they have shown that sparse synchronized fast oscillations can emerge from instabilities of an irregular network dynamics through a Hopf-bifurcation. The emergent oscillation frequency is then inversely proportional to the synaptic delays, which results in a fast rhythm ≥200 Hz. This could be lowered by considering synaptic dynamics. Here we considered the same oscillatory instability condition in equation (6) to derive the stability boundary of the irregular steady state, but developed an alternative framework, the adaptive threshold framework, rather than the Fokker-Planck treatment, to explicitly calculate the necessary transfer functions. One of the transfer functions, rate response function, has been calculated in the presence of subthreshold oscillations for the integrate-and-fire model driven by white noise by Richardson *et al.*^21^ A key result of this study is that fluctuation-driven but not mean activity can unmask the subthreshold oscillations in the spike firing patterns of leaky integrator neurons. However, this study considered only isolated neurons rather than an interconnected network and limited its noise statistics to the white noise case. Both of these assumptions could significantly affect the response characteristics as it has been shown for leaky integrate-and-fire neurons^36^. However, extending the Fokker-Planck framework in the presence of coloured noise and subthreshold resonance is analytically hard and has so far not been attempted to the best of our knowledge. Recent studies have started addressing networks of neurons with a subthreshold frequency preference and rate adaptation; however, the analytical treatment has so far relied on time scale separation between *τ*_*w*_ and *τ*_*V*_, low noise activity and numerical simulations^18^. Yet, our study shows that the regime where *τ*_*w*_ and *τ*_*V*_ interact is particularly interesting, because it is here that rate and subthreshold resonances are the most pronounced. Despite these differences, we can confirm the observation by Augustin *et al.*^18^ that in the absence of adaptive variable *w* even excitation-dominated networks lack resonances at any frequency.

Next, we discuss how our results complement previous studies addressing the effect of gap junctions on network dynamics. Ever since gap junctions were first observed in higher brain regions, theorists began to address the similarities and differences between chemical and electrical synaptic communication. Mostly, these studies considered a low-noise regime where all neurons fire regularly and can be described by phase-coupled oscillators^13^,^14^,^15^. Counterintuitively, these studies have shown that direct voltage coupling through gap junctions does not necessarily lead to network-wide synchronization but, depending on parameters, could also be a desynchronizing force. However, although the regular firing regime assumed in these studies can occur in the periphery, this is not a typical situation for the cortex where coefficient of variation (CV) and Fano values are close to 1 and fluctuations occur rather than the mean drive spiking^26^,^37^. This is a situation where global network oscillations could be difficult to achieve and the synchronizing effect of gap junctions could be cancelled. One of the few studies addressing the stability of the irregular spiking regime in the presence of gap junctions is by Ostojic *et al.*^24^ Studying an inhibitory leaky integrate-and-fire network coupled by gap junctions, Ostojic *et al.* found that network with gap junctions can indeed synchronize in the presence of noise and heterogeneities, and the period of oscillation is determined by the intrinsic firing rate of the neurons. This is in contrast to inhibitory chemically coupled networks where the oscillatory period is determined by the time course of the inhibitory currents following a spike. Importantly, the authors did not consider the subthreshold frequency preference of inhibitory neurons and neglected the synaptic time constants by assuming a white noise drive. Both of these phenomena can significantly alter the transfer rate function *R*_*i*_(*ω*) and thereby affect the stability and oscillatory properties of networks^38^.

Here we proposed an analytical framework to incorporate these phenomena in a network of neurons connected by electrical and chemical synapses, where inhibitory neurons express subthreshold resonance. We show that in the fluctuation-driven regime, inhibitory subthreshold resonance is the substrate for firing resonance of single neurons as well as global oscillations. From a fluctuation-driven regime, self-sustained oscillations only emerge if the inhibitory neurons have subthreshold resonance and are connected by gap junctions, leading to a net spike-induced depolarizating current. Our model therefore indicates that excitatory neurons that typically lack subthreshold resonance are not essential for global rhythm generation but rather amplify self-sustained oscillations. Our theory predicts that this resonance is amplified by chemical connectivity in the network and gap-junction-mediated subthreshold current, but the resonance peak location is not affected by the connectivity. Our framework nicely accounts for experimental evidences^22^ showing amplification of *γ*-frequencies by periodic stimulation of the inhibitory neurons and of low frequencies by stimulation of excitatory neurons. Thus, our study provides a very-much-needed mechanistic understanding of experimental evidence showing the special role of subthreshold resonance for defining the preferred global oscillation frequency of noise-driven networks as well as in defining the response profile of networks to external stimulation.

## Methods

We consider a network of *N*=*N*_E_+*N*_I_ neurons, where *N*_I_ is the number of inhibitory and *N*_E_ is the number of excitatory neurons. Each neuron is modelled by an integrate-and-fire model without reset and an additional adaptation variable, and simulated for a time *T*_sim_ with a resolution of dt=0.01 ms. The code used to simulate the model will be posted on ModelDB ( https://senselab.med.yale.edu/modeldb).

### Dynamics of a single neuron

We consider a single neuron that exhibits a subthreshold frequency preference and describe the voltage at cell *j* by the following two dimensional differential equations,


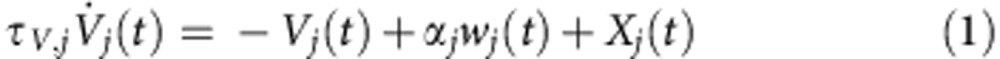



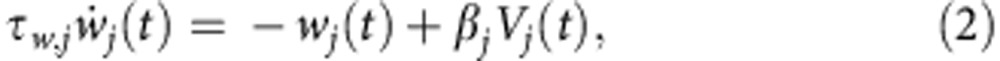


where *τ*_*V,j*_ and *τ*_*w*,*j*_ are the time constant of the voltage and the adaptation variable respectively, *X*_*j*_(*t*) is the current, *α*_*j*_ and *β*_*j*_ are coupling constants. *j*ε{*E*, *I*} where E and I represents the excitatory and inhibitory neurons, respectively. The parameters for both populations are summarized in Table 1. We note that this simple leaky integrator dynamics with one adaptation variable *w* is a linearized representation of a physiologically more accurate conductance-based Hodgkin–Huxley^21^. A spike is emitted by the neuron *j*, whenever *V*_*j*_(*t*) crosses the voltage threshold *V*_*th,j*_ from below^39^,^40^,^41^,^42^. This reset-free spike implementation is compatible to the classical integrate-and-fire model for low rates and finite time constants^41^, and has been shown to capture essential features of spike correlations and response dynamics of cortical neurons in the noise-driven regime^39^,^43^. The subthreshold voltage dynamics in equation (1) is inspired by four recent publications^39^,^40^,^41^,^44^. It can be viewed as a minimal model that due to the adaptation variable *w*_*i*_ can exhibit subthreshold resonance. Although there are a number of cellular mechanisms other than adaptation that can contribute to a subthreshold frequency preference^45^,^46^, for reasons of mathematical tractability we opted for the above implementation. The subthreshold resonance emerges in equation (1) if the eigenvalues of the two-dimensional transformation are complex, see Fig. 1a. The subthreshold frequency is determined by the imaginary part of the eigenvalues and grows when the membrane time constant *τ*_*V*_ decreases, see Fig. 1c.

To show the robustness of our results across spiking models, we compare the threshold model with the aEIF model^25^, where the voltage *V*_*j*_ at cell *j* and the adaptation variable *w*_*j*_ are described by the following two-dimensional differential equations 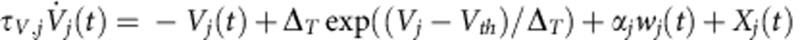 and 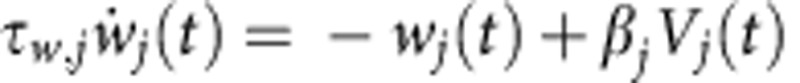, where Δ_*T*_=1/2 mV taken from ref. 25. The threshold condition is if *V*_*j*_(*t*_*j*_)≥*V*_th_, then *V*_*j*_(*t*)=0, ∀*t*ε[*t*_*j*_, *t*_*j*_+*τ*_ref_], *τ*_ref_=5 ms, *w*_*j*_(*t*)=*w*_*j*_(*t*)+const, const=30 mV. We find that all our model predictions can also be observed in the aEIF model and features such as firing rate response, response dynamics and network stability regimes are preserved across models, see Figs 2c and 3, and Supplementary Figs 1 and 2.

### Single-neuron response to dynamical stimuli

To address how interconnected networks respond to dynamical stimuli, the first step is to understand how the firing rate of a single neuron responds to weak sinusoidal current drive of frequency *f* (refs 19, 21). We therefore derive the linear response function *R*_*j*_(*ω*) for a neuron *j*ε[*E*, *I*]


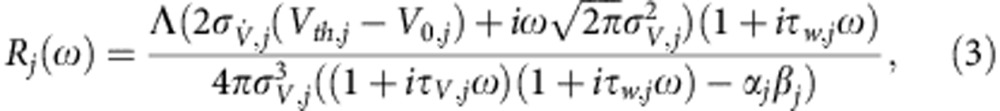


where *ω*=2*πf*, *V*_0,*j*_ is the mean voltage of the neuron *j*, 
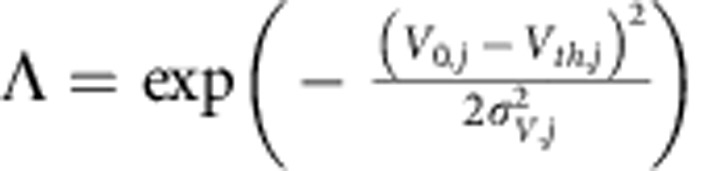
, *σ*_*V,j*_ is the variance of *V*_*j*_ (derived in equations (15–21) in the Supplementary Methods). All other parameters as in equation (1) and their values for excitatory and inhibitory neurons are summarized in Table 1. We choose the parameter set for both neurons to match the adaptation and membrane time constants, rate resonance peak and the reliably encoded frequency range that are observed experimentally in cortical neurons^6^,^19^,^43^. The reverse characteristic transfer function of a single neuron *j* is the current response function *S*_*j*_(*ω*) that governs how the average input current of single neuron responds to weak sinusoidal rate drive of frequency *f*.


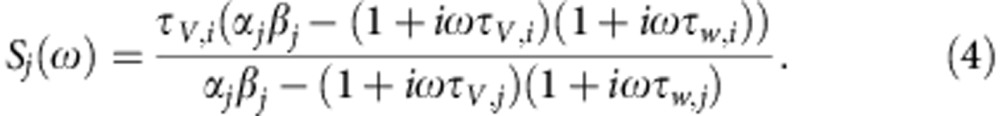


Detailed derivation of *R*_*j*_(*ω*) and *S*_*j*_(*ω*), as well as their generalization for electrically connected neurons, can be found in the Supplementary Methods. It it is important to emphasize that the linear response function describes the rate response at the applied stimulus frequency *ω*. Even though this linear response is derived mathematically for small inputs, it is also valid for larger input stimuli. Intuitively, we know that strong stimuli at frequency *ω* excite responses at *ω* and higher-order harmonics at *nω*. As the amplitude grows, the amplitude of *nω* components grows too. However, as we are interested in the response at *ω*, the higher-order harmonics, *nω*, are negligible relative to the linear response component at *ω*.

### Gap junctions

Inhibitory interneurons are often connected by both gap junctions and chemical GABAergic synapses^11^. Between the spikes, the gap-junction-mediated coupling, *γ*_c_, is proportional to the difference of voltages between the neurons, see equation (5). Consider two inhibitory neurons chemically and electrically coupled. When a spike arrives at a GABAergic synapse, a brief inhibitory current pulse (IPSC) is triggered in the postsynaptic neuron (see Fig. 3a, bottom) for a schematic depiction. This is the well-known spike transmission at chemical synapses, which we model here via an exponential IPSC Γ_I_*θ*(*t*−*t*_*j*_) exp(−*t*/*τ*_I_) at the time of spike *t*_*j*_. On the other hand, the same spike is also transmitted through the gap junction resulting in a so-called ‘spikelet’ or electrical postsynaptic potential. The spikelet depends on the shape of the presynaptic spike: spikes characterized by a brief depolarization and large hyperpolarization tend to evoke predominantly inhibitory spikelets, while spikes with a prolonged depolarization lead to a primarily excitatory spikelets. A number of studies have investigated the spikelet shape and reported a great diversity^47^,^48^. For example, parvalbumin-positive interneurons in the somatosensory cortex in L2/3, the gap junction evoked spikelets are purely excitatory^47^, while in the amygdala the spikelet of parvalbumin-positive interneurons can have a late hyperpolarizing transient^49^. In this paper, we assume that the spikelet contribution is excitatory, although our framework in not limited to that. Mathematically, we model the spikelet as an exponential at the time of spike *t*_*j*_ with the same time constant as the IPSC. We can therefore sum the contribution of the GABAergic IPSC and the spikelet, leading to a net exponential postsynaptic potential Γ_II_*θ*(*t*−*t*_*j*_) exp(−*t*/*τ*_I_). This single exponential approximation serves to keep the analytical complexity at bay while considering the dominant finite time scale of synaptic interactions.

### Dynamics of the network

Interested in the temporal spike dynamics of a network with electrically and chemically coupled inhibitory neurons and only chemically coupled excitatory neurons, we model the input current *X*_*j*_(*t*) to a neuron in the irregular steady state as a sum of chemical and electrical coupling. The topology of the network is all-to-all with the connectivity matrix Γ_*ij*_. The all-to-all connectivity is not critical and we observed equivalent results with sparse connectivity, as long as the product between the connectivity probability and the connectivity strength remains the same. Note that the inhibitory spike-mediated coupling Γ_II_=Γ_E,gap_+Γ_I_ consists of a spikelet-mediated excitatory contribution (Γ_E,gap_) and the contribution of GABAergic chemical synapses (Γ_I_). We represent each spike triggered electrical and chemical synaptic potential by an exponential of the form Γ_*ij*_*θ*(*t*) exp(−*t*/*τ*_I_) such that the external fluctuations *η*_ext_(*t*) are of the Ornstein Uhlenbeck type with time constant *τ*_I_ (ref. 50). We neglect synaptic delays and assume that both chemical and electrical spike interactions are instantaneous. We operate in the irregular steady state where the mean recurrent current drive is proportional to the firing rate^34^. For the sake of tractability, we assume that the fluctuations in the external input *σ*_*I*_ dominate over network-generated noise and are sufficient to describe the firing rate of the network (see Fig. 1d for a comparison between theory and numerical simulations). Explicitly, we model the input current *X*_*j*_(*t*) to a neuron in the irregular steady state as


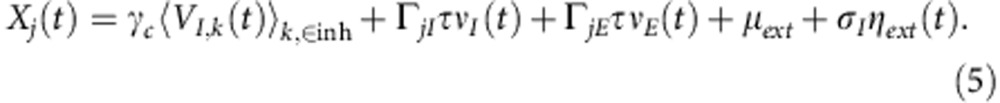


Here, ‹*V*_*I,k*_(*t*)›_*k*,εinh_ is the average voltage of the inhibitory population, *v*_E_(*t*) is the firing rate of the excitatory population, *v*_I_(*t*) is the firing rate of the inhibitory population, *μ*_ext_ is the constant external drive and *σμ*_ext_(*t*) the fluctuating external input. *γ*_c_ is the subthreshold coupling contributed by gap junctions. The strength of excitatory and inhibitory spike triggered interactions for the population *j* are Γ_*j*E_ and Γ_*j*I_, respectively. The parameters are given in Table 1.

### Steady-state firing rate

First, we study the firing rate as a function of current drive in a single, isolated neuron, driven by a mean current *X*_0_=*μ*_ext_+*σ*_I_*η*_ext_ where *η*_ext_ is an Ornstein Uhlenbeck fluctuation with correlation time *τ*_I_ and unit variance. The firing rate *v*_*i*_ is given in the Supplementary Methods and is shown on Fig. 1d. The theoretically derived firing rate *v*_*i*_ as a function of *μ*_ext_/*V*_th_ is in good agreement with numerical simulations. In a mixed irregular, asynchronous network with both excitation and inhibition, the steady-state firing rate of inhibitory neurons *v*_I_ and excitatory neurons *v*_E_ are derived in the Supplementary Methods and shown in the Supplementary Fig. 1c,d, again matching numerical simulations.

### Condition for self-sustained oscillations

To assess the stability of the asynchronous irregular network state with respect to oscillatory perturbations, we consider the stability conditions outlined in refs 23, 29. Starting with an infinitesimal time-dependent perturbation *δ*_I_(*ω*) exp(*iωt*_N_) in current applied at time *t*_N_, we obtain changes in firing rate and resulting changes in current induced by as change in firing rate. For a network consisting of only inhibitory neurons, the stability condition reads


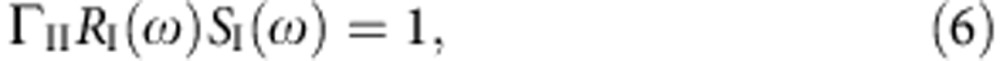


where Γ_II_ is the recurrent connectivity strength, and *R*_I_ and *S*_I_ are response functions given in equation (3) and equation (4), respectively. For the general two-dimensional network with an excitatory and inhibitory subpopulations, the stability condition takes a matrix form and is provided in the Supplementary Methods. Studying equation (6) and its real part, we find that for all Γ_II_ <0 no real *ω* solution can be obtained implying that the irregular regime is stable for inhibitory interactions. Another condition for the stability of the irregular steady state is obtained if subthreshold resonance is lacking (*α*=0). In this case we find


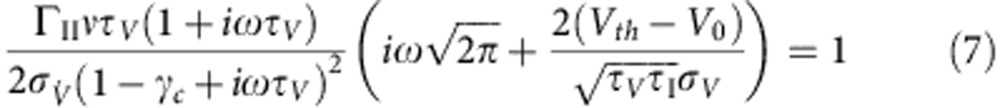


Solving for *ω* we obtain only imaginary solutions implying that the oscillatory instabilities can not emerge and the irregular steady state is stable for any Γ_II_ under these conditions. Although this condition is derived for the threshold neuron model, we show in the Supplementary Fig. 2 that it also holds for the adaptive leaky and aEIF neurons with reset.

### Quantification of global network coherence

In simulations, we assess the global network coherence using the rate-normalized correlation *ρ*. For each excitatory (E) and inhibitory (I) population, we compute the correlation according to 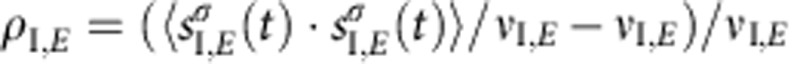. Here, ‹·› denotes the ensemble average, *v*_I,E_ are the rates of excitatory and inhibitory neurons, respectively. *s*_I,E_ are the compound spike trains of the respective populations. To compute the spike correlation 
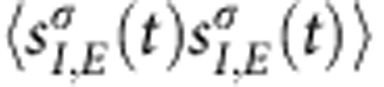
, we convolve each spike with a Gaussian kernel with *σ*=3 ms, leading to 
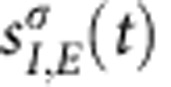
. If the network is in an asynchronous, irregular state, then we expect *ρ*=0. In contrast, if the network is in an oscillatory state with each spike aligned across the population then lim_*σ*→0_ *ρ*=∞. We therefore use *ρ*_I,E_ as an index of population synchrony. In a mixed excitation-inhibition network we use the average Cor=(*ρ*_*E*_+*ρ*_*I*_)/2 as a measure of network coherence 51, for example in Fig. 4a. Note that in practice in the asynchronous irregular regime *ρ* will fluctuate around zero, with the amplitude of fluctuations decreasing for growing network size *N*.

## Author contributions

T.T. and C.C. planned the research, analysed the data, performed the analytical and numerical computations and wrote the paper.

## Additional information

**How to cite this article:** Tchumatchenko, T. and Clopath, C. Oscillations emerging from noise-driven steady state in networks with electrical synapses and subthreshold resonance. *Nat. Commun.* 5:5512 doi: 10.1038/ncomms6512 (2014).

## Supplementary Material

Supplementary Info Supplementary Figures 1-4 and Supplementary Methods

## Figures and Tables

**Table 1:** Parameter sets for excitatory and inhibitory neurons.

**Neuron type**	***V***_**th,i**_	***σ***_**I,i**_	***τ***_**I,i**_	***α***_**i**_	***β***_**i**_	***τ***_**V,i**_	***τ***_**w,i**_	***μ***_**E/I**_
Excitatory	10 mV	12 mV	10 ms	0	—	40	—	0.5*V*_th,E_
Fig. 3f,g		−50%						−30*V*_th,E_
InhibitoryFigs 2c and 3Fig. 3f,g	4 mV	10 mV30 mV−50%	10 ms	−2	4.5	10	20	2*V*_th,I_−4 *V*_th,E_ −30*V*_th,I_
**Network**	***γ*_c_**	**Γ_II_**	**Γ_EI_**	**Γ_IE_**	**Γ_EE_**	***N***	***N*_E_/*N*_I_**	***T*_sim_**
Figs 2 and 3Fig. 4	0.5	15 mV	−10 mV	15 mV	15 mV	5005000	0.8/0.2	10^3^ s5 s

## References

[b1] Wang X.-J. Neurophysiological and computational principles of cortical rhythms in cognition. Physiol. Rev. 90, 1195–1268 (2010). 20664082 10.1152/physrev.00035.2008 PMC2923921

[b2] Melloni L. *et al.* Synchronization of neural activity across cortical areas correlates with conscious perception. J. Neurosci. 27, 2858–2865 (2007). 17360907 10.1523/JNEUROSCI.4623-06.2007 PMC6672558

[b3] Zijlmans M., Jacobs J., Zelmann R., Dubeau F. & Grotman J. High-frequency oscillations mirror disease activity in patients with epilepsy. Neurology 72, 979–986 (2009). 19289737 10.1212/01.wnl.0000344402.20334.81 PMC3797085

[b4] Sohal V. S., Zhang F., Yizhar O. & Deisseroth K. Parvalbumin neurons and gamma rhythms enhance cortical circuit performance. Nature 459, 698–702 (2009). 19396159 10.1038/nature07991 PMC3969859

[b5] Hutcheon B. & Yarom Y. Resonance, oscillation and the intrinsic frequency preferences of neurons. Trends Neurosci. 23, 216–222 (2000). 10782127 10.1016/s0166-2236(00)01547-2

[b6] Pike F. G. *et al.* Distinct frequency preferences of different types of rat hippocampal neurones in response to oscillatory input currents. J. Physiol. 529, 205–213 (2000). 11080262 10.1111/j.1469-7793.2000.00205.x PMC2270176

[b7] Fellous J. -M. *et al.* Frequency dependence of spike timing reliability in cortical pyramidal cells and interneurons. J. Neurophysiol. 85, 1782–1787 (2001). 11287500 10.1152/jn.2001.85.4.1782

[b8] Tateno T., Harsch A. & Robinson H. P. C. Threshold firing frequency-current relationships of neurons in rat somatosensory cortex: Type 1 and type 2 dynamics. J. Neurophysiol. 92, 2283–2294 (2004). 15381746 10.1152/jn.00109.2004

[b9] Mamor Y., Rinzel J., Segev I. & Yarom Y. Low-amplitude oscillations in the inferior olive: A model based on electrical coupling of neurons with heterogeneous channel densities. J. Neurophysiol. 77, 2736–2752 (1997). 9163389 10.1152/jn.1997.77.5.2736

[b10] Deans M. R., Gibson J. R., Sellitto C., Connors B. W. & Paul D. L. Synchronous activity of inhibitory networks in neocortex requires electrical synapses containing connexin36. Neuron 31, 477–485 (2001). 11516403 10.1016/s0896-6273(01)00373-7

[b11] Galarreta M. & Hestrin. S. A network of fast-spiking cells in the neocortex connected by electrical synapses. Nature 402, 72–75 (1999). 10573418 10.1038/47029

[b12] Gibson J. R., Beierlein M. & Connors B. W. Functional properties of electrical synapses between inhibitory interneurons of neocortical layer. J. Neurophysiol. 93, 467–480 (2005). 15317837 10.1152/jn.00520.2004

[b13] Pfeuty B., Mato G., Golomb D. & Hansel D. Electrical synapses and synchrony: the role of intrinsic currents. J. Neurosci. 23, 6280–6294 (2003). 12867513 10.1523/JNEUROSCI.23-15-06280.2003 PMC6740557

[b14] Kopell N. & Ermentrout B. Chemical and electrical synapses perform complementary roles in the synchronization of interneuronal networks. Proc. Natl Acad. Sci. USA 101, 15482–15487 (2004). 15489269 10.1073/pnas.0406343101 PMC524455

[b15] Chow C. C. & Kopell N. Dynamics of spiking neurons with electrical coupling. Neural. Comp. 12, 1643–1678 (2000). 10.1162/089976600300015295 10935921

[b16] Lewis T. J. & Rinzel J. Dynamics of spiking neurons connected by both inhibitory and electrical coupling. J. Comput. Neurosci. 14, 283–309 (2003). 12766429 10.1023/a:1023265027714

[b17] Sherman A. & Rinzel J. Rhythmogenic effects of weak electrotonic coupling in neuronal models. PNAS 89, 62471–62474 (1992). 10.1073/pnas.89.6.2471 PMC48680 1549611

[b18] Augustin M., Ladenbauer J. & Obermayer K. How adaptation shapes spike rate oscillations in recurrent neuronal networks. Front. Comput. Neurosci. 7, 9 (2013). 23450654 10.3389/fncom.2013.00009 PMC3583173

[b19] Brunel N., Hakim V. & Richardson M. J. E. Firing-rate resonance in a generalized integrate-and-fire neuron with subthreshold resonance. Phys. Rev. E 67, 051916 (2003). 10.1103/PhysRevE.67.051916 12786187

[b20] Vierling-Claassen D., Cardin J. A., Moore C. I. & Jones S. R. Computational modeling of distinct neocortical oscillations driven by cell-type selective optogenetic drive: separable resonant circuits controlled by low-threshold spiking and fast-spiking interneurons. Front. Hum. Neurosci. 4, 198 (2010). 21152338 10.3389/fnhum.2010.00198 PMC2996257

[b21] Richardson M. J. E., Brunel N. & Hakim V. From subthreshold to firing-rate resonance. J. Neurophysiol. 89, 2538–2554 (2003). 12611957 10.1152/jn.00955.2002

[b22] Cardin J. A. *et al.* Driving fast-spiking cells induces gamma rhythm and controls sensory responses. Nature 459, 663–667 (2009). 19396156 10.1038/nature08002 PMC3655711

[b23] Brunel N. & Hakim V. Sparsely synchronized neuronal oscillations. Chaos 18, 015113 (2008). 18377094 10.1063/1.2779858

[b24] Ostojic S., Brunel N. & Hakim V. Synchronisation properties of networks of electrically coupled neurons in the presence of noise and heterogeneities. J. Comp. Neurosci. 26, 369–392 (2009). 10.1007/s10827-008-0117-3 19034642

[b25] Naud R., Marcille N., Clopath C. & Gerstner W. Firing patterns in the adaptive exponential integrate-and-fire model. Biol. Cybern. 99, 335–347 (2008). 19011922 10.1007/s00422-008-0264-7 PMC2798047

[b26] Shadlen M. N. & Newsome W. T. The variable discharge of cortical neurons: implications for connectivity, computation, and information coding. J. Neurosci. 18, 3870–3896 (1998). 9570816 10.1523/JNEUROSCI.18-10-03870.1998 PMC6793166

[b27] Tiesinga P. & Sejnowski T. J. Cortical enlightenment: are attentional gamma oscillations driven by ING or PING? Neuron 63, 727–732 (2009). 19778503 10.1016/j.neuron.2009.09.009 PMC2778762

[b28] Margrie T. W., Brecht M. & Sakmann B. In vivo, low-resistance, whole-cell recordings from neurons in the anaesthetized and awake mammalian brain. Pflugers Arch. 444, 491–498 (2002). 12136268 10.1007/s00424-002-0831-z

[b29] Brunel N. & Hakim V. Fast global oscillations in networks of integrate-and-fire neurons with low firing rates. Neural Comput. 11, 1621–1671 (1999). 10490941 10.1162/089976699300016179

[b30] Brette R. & Gerstner W. Adaptive exponential integrate-and-fire model as an effective description of neuronal activity. J. Neurophysiol. 94, 3637–3642 (2005). 16014787 10.1152/jn.00686.2005

[b31] Naud R., Marcille N., Clopath C. & Gerstner W. Firing patterns in the adaptive exponential integrate-and-fire model. Biol. Cybern. 99, 335–347 (2008). 19011922 10.1007/s00422-008-0264-7 PMC2798047

[b32] Clopath C., Jolivet R., Rauch A., Luescher H.-R. & Gerstner W. Predicting neuronal activity with simple models of the threshold type: adaptive exponential integrate-and-fire model with two compartments. Neurocomputing 70, 1668–1673 (2007).

[b33] Loewenstein Y., Yarom Y. & Sompolinsky. H. The generation of oscillations in networks of electrically coupled cells. PNAS 98, 8095–8100 (2001). 11427705 10.1073/pnas.131116898 PMC35473

[b34] van Vreeswijk C. A. & Sompolinsky H. Chaotic balanced state in a model of cortical circuits. Neural Comp. 10, 1321–1371 (1998). 10.1162/089976698300017214 9698348

[b35] Brunel N. & Wang X.-J. What determines the frequency of fast network oscillations with irregular neural discharges? J. Neurophysiol. 90, 415–430 (2003). 12611969 10.1152/jn.01095.2002

[b36] Brunel N., Chance F. S., Fourcaud N. & Abbott L. F. Effects of synaptic noise and filtering on the frequency response of spiking neurons. Phys. Rev. Lett. 86, 2186–2189 (2001). 11289886 10.1103/PhysRevLett.86.2186

[b37] Kara P., Reinagel P. & Reid R. Low response variability in simultaneously recorded retinal, thalamic, cortical neurons. Neuron 27, 635–646 (2000). 11055444 10.1016/s0896-6273(00)00072-6

[b38] Chance F. S., Abbott L. & Reyes A. Gain modulation from background synaptic input. Neuron 35, 773–782 (2002). 12194875 10.1016/s0896-6273(02)00820-6

[b39] Tchumatchenko T., Malyshev A., Geisel T., Volgushev M. & Wolf F. Correlations and synchrony in threshold neuron models. Phys. Rev. Lett. 104, 058102 (2010). 20366796 10.1103/PhysRevLett.104.058102

[b40] Burak Y., Lewallen S. & Sompolinsky H. Stimulus-dependent correlations in threshold-crossing spiking neurons. Neural Comput. 21, 2269–2308 (2009). 19409055 10.1162/neco.2009.07-08-830

[b41] Badel L. Firing statistics and correlations in spiking neurons: A level-crossing approach. Phys. Rev. E 84, 041919 (2011). 10.1103/PhysRevE.84.041919 22181187

[b42] Tchumatchenko T., Geisel T., Volgushev M. & Wolf F. Signatures of synchrony in pairwise count correlations. Front. Comput. Neurosc.i 4, 1 (2010). 10.3389/neuro.10.001.2010 PMC2857958 20422044

[b43] Tchumatchenko T., Malyshev A., Wolf F. & Volgushev M. Ultra-fast population encoding by cortical neurons. J. Neurosci. 31, 12171–12179 (2011). 21865460 10.1523/JNEUROSCI.2182-11.2011 PMC4225046

[b44] Touzel M. P., Monteforte M. & Wolf F. Features of chaotic activity in a balanced network of type II neuronal oscillators. BMC Neurosci. 13, (Suppl 1): P10 (2012).

[b45] Huguenard J. R. & Prince D. A novel t-type current underlies prolonged *Ca*^2+^-dependent burst firing in GABAergic neurons of rat thalamic reticular nucleus. J. Neurosci. 12, 3804–3817 (1992). 1403085 10.1523/JNEUROSCI.12-10-03804.1992 PMC6575965

[b46] Lampl I. & Yarom Y. Subthreshold oscillations and resonant behavior: two manifestations of the same mechanism. Neuroscience 78, 325–341 (1997). 9145790 10.1016/s0306-4522(96)00588-x

[b47] Tamás G., Buhl E. H., Lörincz A. & Somogyi P. Proximally targeted GABAergic synapses and gap junctions synchronize cortical interneurons. Nat. Neurosci. 3, 366–371 (2000). 10725926 10.1038/73936

[b48] Connors B. W. & Long M. A. Electrical synapses in the mammalian brain. Annu. Rev. Neurosci. 27, 393–418 (2004). 15217338 10.1146/annurev.neuro.26.041002.131128

[b49] Woodruff A. R. & Sah P. Networks of parvalbumin-positive interneurons in the basolateral amygdala. J. Neurosci. 27, 553–563 (2007). 17234587 10.1523/JNEUROSCI.3686-06.2007 PMC6672782

[b50] Destexhe A., Rudolph M. & Pare D. The high-conductance state of neocortical neurons *in vivo*. Nat. Rev. Neurosci. 4, 739–751 (2003). 12951566 10.1038/nrn1198

[b51] Buzsaki G., Anastassiou C. A. & Koch C. The origin of extracellular fields and currents - EEG, ECoG, LFP and spikes. Nat. Rev. Neurosci. 13, 407–420 (2012). 22595786 10.1038/nrn3241 PMC4907333

