# Supplementary material for: Oscillations emerging from noise-driven steady state in networks with electrical synapses and subthreshold resonance
Source: Nat Commun. 2014 Nov 18;5:5512. doi: 10.1038/ncomms6512 (PMC4243246; doi:10.1038/ncomms6512)
Supplement: Supplementary Info — Supplementary Figures 1-4 and Supplementary Methods [file ncomms6512-s1.pdf]

## Supplementary Figures

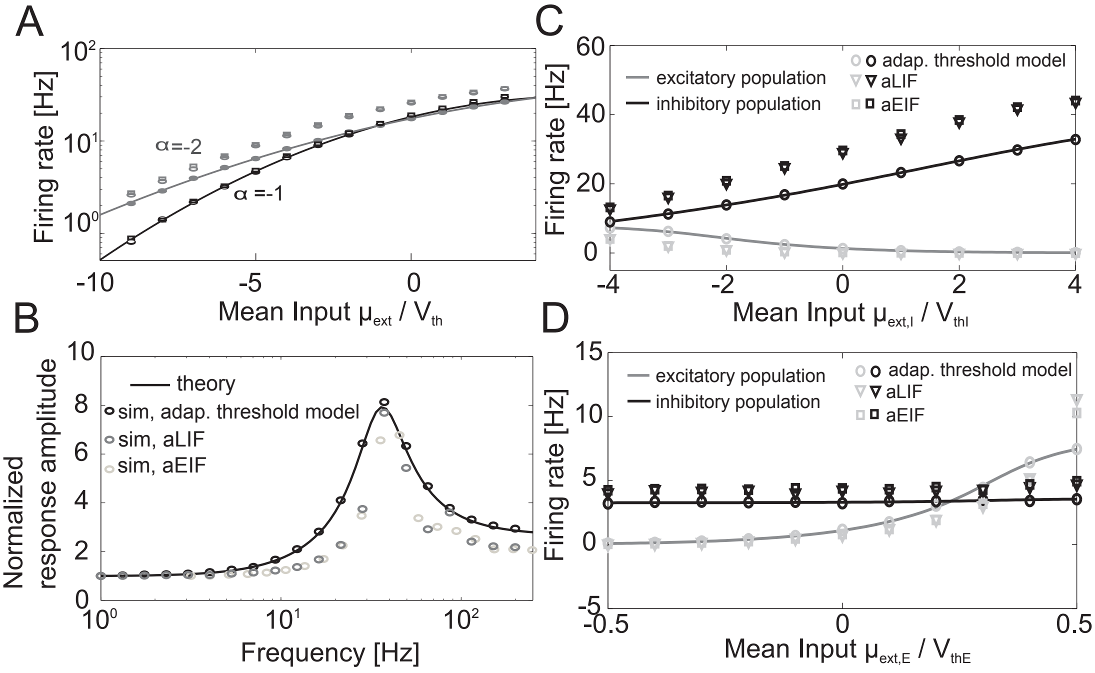


Supplementary Figure 1: **Voltage reset preserves the firing rate dependence on current** (A) Firing rate of a single neuron as a function of current for different adaptation currents ($\alpha$). Solid lines denote the theoretical prediction in Eq. 7 in the main text, filled circles denote the numerical simulations of the threshold crossing model, and empty circles denote the equivalent simulations considering the voltage reset after each spike. (B) Linear rate response amplitude as a function of input frequency in the presence of adaptation ($\alpha = -2$, other parameters as in the inhibitory neurons in the main text). Solid black line indicates the theoretical prediction in Eq. 11 in the main text; solid circles denote the corresponding threshold neuron simulations, and empty circles denote the corresponding simulations that include a voltage reset. (C-D) Network firing rates of excitatory (gray) and inhibitory (black) neurons as a function of inhibitory (C) and excitatory (D) external current. Solid lines denote the theoretical predictions in Eq. 8 and in the main text, empty circles denote the numerical simulations of the threshold crossing model, and filled circles denote the equivalent simulations considering the voltage reset after each spike.

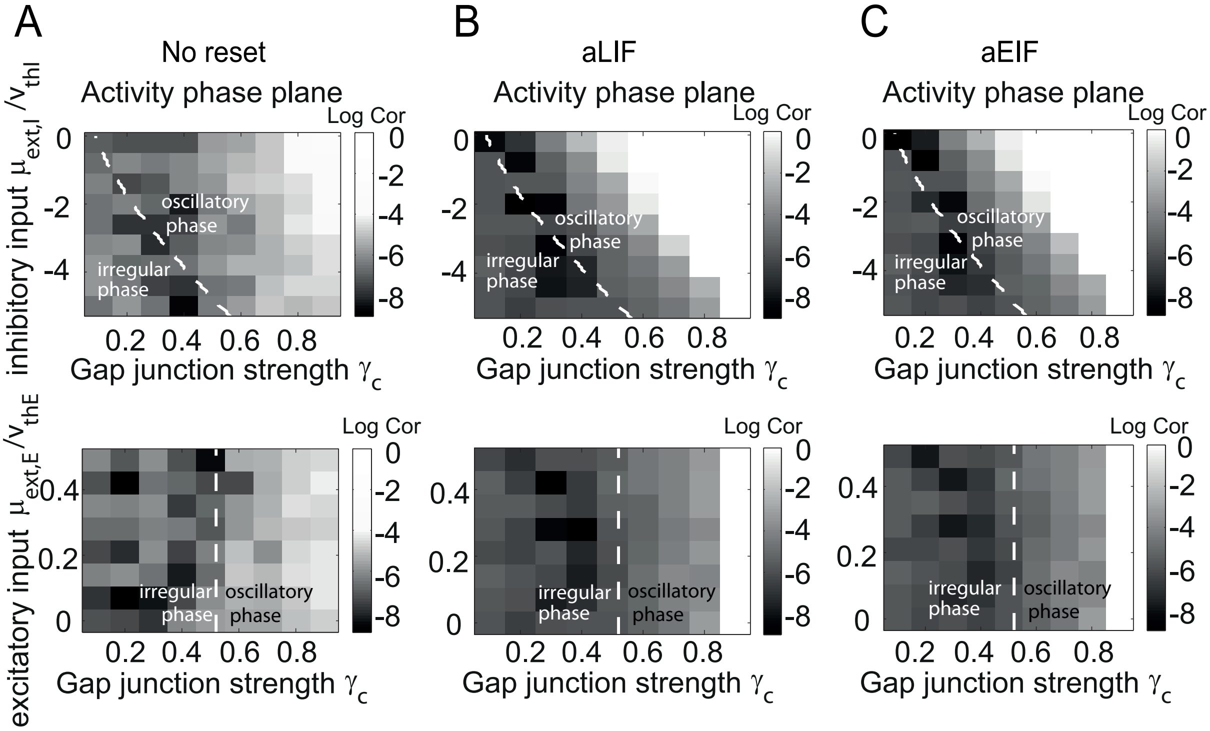


Supplementary Figure 2: **Subthreshold dynamics rather than spike triggered currents define the phaseboundary between irregular and oscillatory network states** (A) Stability regimes of threshold neuron networks with subthreshold frequency preference as a function of gap-junction and inhibitory current drive, white dashed line indicates the predicted phase boundary in this model. (B) Stability regimes of the corresponding adaptive leaky integrate and fire model with subthreshold frequency preference (includes voltage reset and spike triggered adaptation currents, see equations above), white dashed line indicates the predicted phase boundary from (A). (C) Stability regimes of an adaptive exponential integrate and fire model with subthreshold frequency preference (includes voltage reset and spike triggered adaptation currents, see equations above), white dashed line indicates the predicted phase boundary from (A). Color code denotes logarithm of the peak network averaged pairwise spike correlation. Solid black line indicates the phase transition between irregular and oscillatory state in Eq. 5 in the main text. The three models give similar results as long as the adaptation time constant is larger than the membrane time constant as seen experimentally.

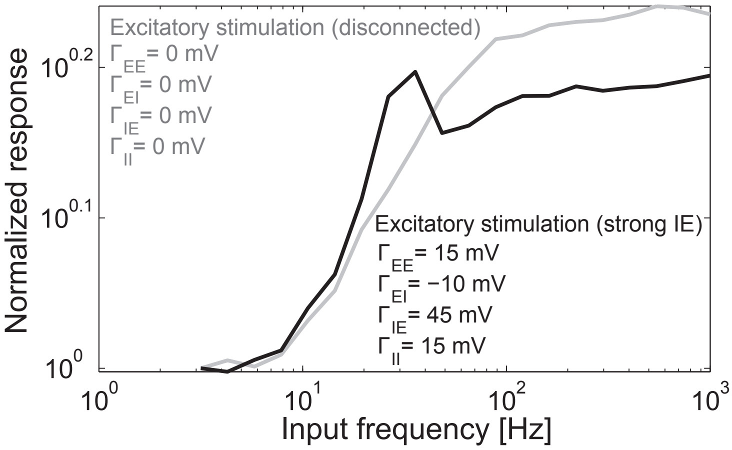


Supplementary Figure 3: **Network response to excitatory stimulation can carry signatures of inhibitory resonance** Network firing rate response to stimulation of excitatory neurons as a function of input frequency in a disconnected network (gray), and in a network with strong E to I connections (black), which reveals signatures of inhibitory resonance (at 40 Hz); connectivity as in the figure legend. In both panels $N = 500$ neurons, $N_E/N_I = 0.8/0.2$, $\gamma_c = 0.5$, $\tau_{V,E} = 5$ ms, all others in Tab. 1 in the main text.

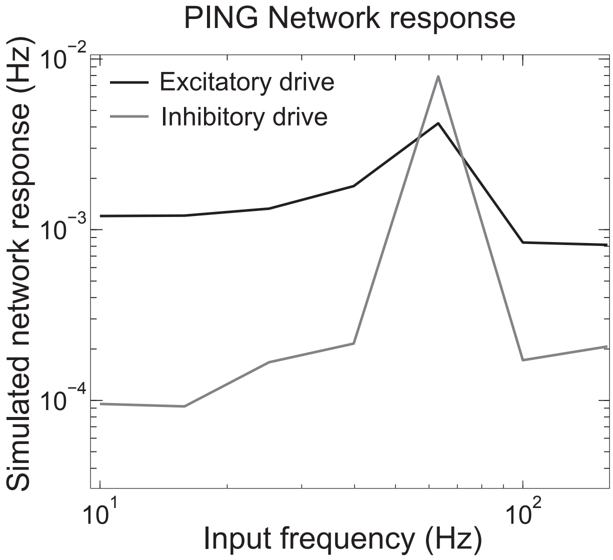


Supplementary Figure 4: **PING network's response external stimulation** Network firing rate response to stimulation of excitatory neurons (black) or inhibitory neurons (gray) as a function of input frequency in a mean-driven network. The network consists of inhibitory neurons without subthreshold resonance ($\alpha = 0$), nor gap-junctions $\gamma_c = 0$. For both population, the spiking thresholds are $V_0 = 10$mV, the time constants are $\tau_I = 40$ms, and external drive $\mu_{ext} = 4\ V_0$, and the noise $\sigma_I = 2$mV. Finally, the network is weakly coupled chemically, $\Gamma_{EE} = \Gamma_{IE} = 4$mV, $\Gamma_{EI} = \Gamma_{II} = -4$mV. 5000 neurons were simulated for 10s, with a cosine amplitude of 0.5 $V_0$. Similar results on PING networks which exhibit a resonant at $\gamma$ response when the excitatory cells are stimulated were previously reported by Tiesinga and Sejnowski in 2009.

# Supplementary Methods

### Network model derivation and numerical implementation

We numerically implement the voltage dynamics at a neuron $i$ via a two dimensional set of equations, that are derived from standard current based spiking models.

$$c_m \dot{V}_i = - g_m V_i + \frac{\Gamma_c \delta_{i,I}}{N_I} \sum_{j \neq i} (V_j - V_i) + \alpha_w w_i(t) + \frac{\Gamma_{iE} c_m}{N_E} \sum_{j \in \text{exc}} \sum_{n_j=-\infty}^{\infty} \delta(t - t_{n,j}) \quad (1)$$

$$+ \frac{\Gamma_{iI} c_m}{N_I} \sum_{j \in \text{inh}} \sum_{n_j=-\infty}^{\infty} \delta(t - t_{n,j}) + I_{ext}(t) + \Sigma \eta_{ext}(t) \quad (2)$$

$$\tau_w \dot{w}_i(t) = -w_i(t) + \beta V_i(t) \quad (3)$$

where $c_m$ is the membrane capacitance, $g_m$ the membrane conductance, $\Gamma_c$ gap-junction conductance, $\alpha_w$ adaptation coupling and $\Gamma_{iE}$ and $\Gamma_{iI}$ are the strengths of the excitatory and inhibitory chemical synapses for the neuron $i$, respectively. $\delta_{i,I}$ is non-zero only for inhibitory neurons, $\delta_{i,I} = 1$ if the neuron $i$ is inhibitory else $\delta_{i,I} = 0$. For tractability, we chose to the same gap-junction strength $\Gamma_c$ for all inhibitory neurons in the analytical steady steady calculations. Furthermore, we consider a two dimensional chemical connectivity matrix $((\Gamma_{EE}, \Gamma_{EI}), (\Gamma_{IE}, \Gamma_{II}))$, where an entry $\Gamma_{ij}$ denotes the chemical coupling for a neuron in the population $i$ to any neuron in the population $j$, $i, j \in (E, I)$.
We use the following abbreviations $\tau_V = \frac{c_m}{g_m+\Gamma_c}$, $\gamma_c = \frac{\Gamma_c}{g_m+\Gamma_c}$, $\alpha = \frac{\alpha_w}{g_m+\Gamma_c}$, $\mu_{ext}(t) = \frac{I_{ext}(t)}{g_m+\Gamma_c}$, $\sigma_I = \frac{\Sigma}{g_m+\Gamma_c}$, $\nu_E(t) = \frac{\sum_{j\in\text{exc}} \sum_{n_j=-\infty}^{\infty} \delta(t-t_{n,j})}{N_E}$, $\nu_I(t) = \frac{\sum_{j\in\text{inh}} \sum_{n_j=-\infty}^{\infty} \delta(t-t_{n,j})}{N_I}$. Together, these abbreviations lead to the short rescalled notation mentioned in the main text

$$\tau_V \dot{V}_i(t) = -V_i(t) + \alpha w_i(t) + X(t) \quad (4)$$

$$X(t) = \gamma_c \delta_{i,I} \langle V_{I,j}(t) \rangle_j + \Gamma_I \tau \nu_I(t) + \Gamma_E \tau \nu_E(t) + \mu_{ext} + \sigma_I \eta_{ext}(t) \quad (5)$$

$$\tau_w \dot{w}_i(t) = -w_i(t) + \beta V_i(t) \quad (6)$$

We note, that the single neuron is described by the following two dimensional dynamics

$$\begin{pmatrix} \dot{V}(t) \\ \dot{w}(t) \end{pmatrix} = \begin{pmatrix} -1/\tau_V & \alpha/\tau_V \\ \beta/\tau_w & -1/\tau_w \end{pmatrix} \begin{pmatrix} V(t) \\ w(t) \end{pmatrix} \text{with eigenvalues} \quad (7)$$

$$\lambda_{1/2} = \frac{-(\tau_V + \tau_w) \pm \sqrt{(\tau_V + \tau_w)^2 + 4\tau_V \tau_w (\alpha\beta - 1)}}{2\tau_V \tau_w} \quad (8)$$

Note that both eigenvalues have a negative real part, for all $\alpha\beta < 1$.

Here, $\langle V_{I,j}(t) \rangle_j$ is the average voltage of the inhibitory population, $\nu_E(t)$ the firing rate of the excitatory population, $\nu_I(t)$ the firing rate of the inhibitory population, $\mu_{ext}$ the constant external drive and $\sigma \mu_{ext}(t)$ the fluctuating external input. Furthermore, we chose the external fluctuations $\eta_{ext}(t)$ to be an Ornstein Uhlenbeck process (OU) with a time constant $\tau_I$ and unit variance. We assign all chemical synapses within the network an exponential post synaptic potential of the form $\exp(-t/\tau_I)$. For the sake of tractability, we also assume that the fluctuations in the external input have a much higher amplitude than those of the recurrently generated firing rates and average inhibitory voltage, such that $var(X) \approx \sigma_I^2$.

## Model generalizations to include spike triggered currents and voltage reset

Our spiking model in Eq. 1 in the main manuscript can be modified to include spike triggered currents and voltage reset. Notably, these modifications leave many predictions we derived in our article intact. Here, we consider two alternative spiking models that include voltage reset, the adaptive leaky integrate and fire (aLIF) and the adaptive exponential integrate and fire (aEIF). We compare the firing rates, response functions and phase transitions derived in these models to the predictions of the reset-free threshold model in Fig. 1,2 in this supplementary material. Fig. 1A,C,D demonstrate that the input-to-firing rate transformation is consistent across these models. Fig. 1B shows that the resonance position is also preserved across these three models. Finally, Fig. 2 shows that the phase boundary between irregular and oscillatory state is preserved across models. Together these findings support the notion that threshold model we put forward provides a useful model substrate to address the dynamical response and stability in live neurons as well as many physiologically important classes of models.

*Adaptive leaky integrate and fire neuron model (aLIF)* We consider a single leaky integrate and fire neuron neuron that exhibits a subthreshold frequency preference and describe the voltage $V_j$ at cell $j$ by the following two dimensional differential equations

$$\tau_{V,j}\dot{V}_j(t) = -V_j(t) + \alpha_j w_j(t) + X_j(t) \tag{9}$$
$$\tau_{w,j}\dot{w}_j(t) = -w_j(t) + \beta_j V_j(t), \tag{10}$$

with a threshold condition

$$\text{if} \quad V_j(t_j) \geq V_{th}, \ \text{then} \begin{cases} V_j(t) = 0, & \forall t \in [t_j, t_j + \tau_{ref}], \tau_{ref} = 5\text{ms} \\ w_j(t) = w_j(t) + const, & const = 30mV. \end{cases} \tag{11}$$

*Adaptive exponential integrate and fire neuron model (aEIF)* We consider a single leaky integrate and fire neuron neuron that exhibits a subthreshold frequency preference and describe the voltage $V_j$ at cell $j$ by the following two dimensional differential equations

$$\tau_{V,j}\dot{V}_j(t) = -V_j(t) + \Delta_T \exp((V_j - V_{th})/\Delta_T) + \alpha_j w_j(t) + X_j(t) \tag{12}$$
$$\tau_{w,j}\dot{w}_j(t) = -w_j(t) + \beta_j V_j(t), \tag{13}$$

where $\Delta_T = 1/2mV$. The threshold condition is

$$\text{if} \quad V_j(t_j) \geq V_{th}, \text{then} \begin{cases} V_j(t) = 0, & \forall t \in [t_j, t_j + \tau_{ref}], \tau_{ref} = 5\text{ms} \\ w_j(t) = w_j(t) + const, & const = 30mV. \end{cases} \tag{14}$$

## Derivation of voltage variance and firing rate in a single neuron

Here we calculate the firing rate of a single model neuron that is described by Eqs. 4 assuming a fluctuation-drive regime. The neuron fires a spike whenever a fixed threshold $V_{th}$ is crossed from below. After the threshold is crossed the voltage follows its original trajectory without reset. Mathematically, the spike times then correspond to upcrossings of a Gaussian potential, and their rate is given by the Rice rate.

$$\nu = \frac{\sigma_{\dot{V}}}{2\pi\sigma_V} \exp(-\frac{(V_0 - V_{th})^2}{2\sigma_V^2}). \tag{15}$$

Below we calculate the three quantities determining the firing rate of a neuron in Eq. 15: the standard deviation of voltage fluctuations $\sigma_V$, the standard deviation of derivative fluctuations $\sigma_{\dot{V}}$ and the threshold distance-to-variance ratio $(V_0 - V_{th})/\sigma_V$. To do so, we will separate $X(t)$ in Eqs. 5 and 4 in a constant and

a fluctuating part, $X(t) = X_0 + \eta(t)$. We aim to calculate the quantities of interest by transforming the Eq. 4 into the Fourier domain and taking advantage of the Wiener Khinchine Theorem. The Fouriermodes are then given by

$$V_i(\omega) = \frac{-(1 + i\tau_w\omega)\eta(\omega)}{(\alpha\beta - (1 + i\tau_V\omega)(1 + i\tau_w\omega))} - \frac{((1 + i\tau_w\omega)X_0)\delta(\omega)\sqrt{2\pi}}{(\alpha\beta - (1 + i\tau_V\omega)(1 + i\tau_w\omega))} \tag{16}$$

$$w_i(\omega) = \frac{-\beta\eta(\omega)}{(\alpha\beta - (1 + i\tau_V\omega)(1 + i\tau_w\omega))} - \frac{\beta X_0\delta(\omega)\sqrt{2\pi}}{(\alpha\beta - (1 + i\tau_V\omega)(1 + i\tau_w\omega))}. \tag{17}$$

The mean voltage and the power spectrum of the fluctuating part are

$$V_0 = \frac{X_0}{1 - \alpha\beta} \tag{18}$$

$$V(\omega)V^*(\omega) = \frac{(1 + i\tau_w\omega)(1 - i\tau_w\omega)\eta(\omega)\eta^*(\omega)}{(-\alpha\beta + (1 + i\tau_V\omega)(1 + i\tau_w\omega))(-\alpha\beta + (1 - i\tau_V\omega)(1 - i\tau_w\omega))} \tag{19}$$

Assuming that $\eta(t)$ is an Ornstein Uhlenbeck process with a power spectrum $\eta(\omega)\eta^*(\omega) = \sigma_I^2 \frac{\sqrt{2/\pi}\tau_I}{1+\tau_I^2\omega^2}$ we obtain the required variances $\sigma_V$ and $\sigma_{\dot{V}}$

$$\begin{aligned}
\sigma_V^2 &= \int_{-\infty}^{\infty} \frac{V(\omega)V^*(\omega)}{\sqrt{2\pi}} d\omega = \frac{\sigma_I^2\tau_I(\tau_I(\tau_V + \tau_w) + \tau_w(\tau_V + \tau_w - \alpha\beta\tau_w))}{(1 - \alpha\beta)(\tau_V + \tau_w)((1 - \alpha\beta)\tau_I^2 + \tau_V\tau_w + \tau_I(\tau_V + \tau_w))} \\
\sigma_{\dot{V}}^2 &= \int_{-\infty}^{\infty} \omega^2 \frac{V(\omega)V^*(\omega)}{\sqrt{2\pi}} d\omega = \frac{\sigma_I^2(\tau_w(\tau_V + \tau_w) + \tau_I(\tau_V + \tau_w - \alpha\beta\tau_w))}{\tau_V(\tau_V + \tau_w)((1 - \alpha\beta)\tau_I^2 + \tau_V\tau_w + \tau_I(\tau_V + \tau_w))}
\end{aligned} \tag{20}$$

Together with Eq. 15, these results determine the firing rate of a single neuron

$$\nu = \sqrt{\frac{\tau_w T_2 + \tau_I T_1}{4\pi^2\tau_V\tau_I\Lambda C_1}} \exp\left(\frac{-\left(\frac{X_0}{1-\alpha\beta} - V_{th}\right)^2 D_1}{2\sigma_I^2\Lambda^2\tau_I C_1}\right) \tag{21}$$

Where $T_1 = \tau_V + \tau_w(1-\alpha\beta)$, $T_2 = \tau_V + \tau_w$, $\Lambda = (1-\alpha\beta)^{-1}$, $C_1 = \tau_I T_2 + \tau_w T_1$, $D_1 = T_2(\tau_I^2 - \Lambda(\tau_V\tau_w + \tau_I T_2))$. The single neuron firing rate in Eq. 21 and its dependence on the adaptation and mean current drive is demonstrated in Fig.1 C in the main manuscript. Note, that for inhibitory neurons coupled by gap-junctions the mean voltage has to fulfill $X_0/(1 - \alpha\beta) = \gamma_c X_0/(1 - \alpha\beta) + \mu_{ext}$.

**Two population network**

We consider a network in the irregular steady state that consists of excitatory and inhibitory neurons. The currents at the inhibitory and excitatory populations are then given by

$$X_I(t) = \gamma_c V_{0,I} + \Gamma_{II}\tau\nu_I(t) + \Gamma_{IE}\tau\nu_E(t) + \mu_{ext,I} + \sigma_I\eta_{ext}(t) \tag{22}$$

$$X_E(t) = \Gamma_{EI}\tau\nu_I(t) + \Gamma_{EE}\tau\nu_E(t) + \mu_{ext,E} + \sigma_I\eta_{ext}(t) \tag{23}$$

where $V_{0,I}$ is the mean voltage in the inhibitory population. Due to the homogeneity of the connectivity within a population, we can use the population and time average interchangeably. The mean voltage at the inhibitory neurons is then equal to its population average $V_{0,I} = \langle V_{j\in\mathrm{inh}}(t)\rangle_j$.

$$V_{0,I} = \frac{\mu_{ext,I} + \Gamma_{II}\tau\nu_I + \Gamma_{IE}\tau\nu_E}{1 - \alpha_I\beta_I - \gamma_c} \tag{24}$$

$$V_{0,E} = \frac{\mu_{ext,E} + \Gamma_{EI}\tau\nu_I + \Gamma_{EE}\tau\nu_E}{1 - \alpha_E\beta_E} \tag{25}$$

With the mean voltages in each population known from Eq. 24, we can address the self-consistent conditions that the firing rates $\nu_I$ and $\nu_E$ have to meet in the irregular steady state. Considering Eq. 21 that defines the firing rate for a given mean current and the mean currents in Eqs. 24 and 25 that in turn define the mean rates, we derive the self-consistent solution

$$\nu_I = \sqrt{\frac{\tau_{w,I}T_{2I} + \tau_{I,I}T_{1I}}{4\pi^2\tau_{V,I}\tau_{I,I}\Lambda_I C_{1,I}}} \exp\left(\frac{-\left(V_{0,I} - V_{th,I}\right)^2 D_{1,I}}{2\sigma_{I,I}^2\Lambda_I^2\tau_{I,I}C_{1,I}}\right) \tag{26}$$

$$\nu_E = \sqrt{\frac{\tau_{w,E}T_{2E} + \tau_{I,E}T_{1E}}{4\pi^2\tau_{V,E}\tau_{I,E}\Lambda_E C_{1,E}}} \exp\left(\frac{-\left(V_{0,E} - V_{th,E}\right)^2 D_{1,E}}{2\sigma_{I,E}^2\Lambda_E^2\tau_{I,E}}\right) \tag{27}$$

Where $T_{1i} = \tau_{V,i} + \tau_{w,i}(1 - \alpha_i\beta_i)$, $T_{2i} = \tau_{V,i} + \tau_{w,i}$, $\Lambda_i = (1 - \alpha_i\beta_i)^{-1}$, $C_{1,i} = \tau_{I,i}T_{2i} + \tau_{w,i}T_{1i}$, $D_{1,i} = T_{2i}(\tau_{I,i}^2 - \Lambda_i(\tau_{V,i}\tau_{w,i} + \tau_{I,i}T_{2i}))$ where $i = I$ or $E$. The solution to these equations as a function of external currents $\mu_I$, $\mu_E$ is displayed in Fig. 1.

### Derivation of linear transfer functions $R$ and $S$

To study the stability of this steady state we consider the changes in firing rate induced by a change in current, and the changes in current induced by a change in firing rate. Applied sequentially, they lead to the iterative function that needs to be stable:

$$I(t_{N+1}) = I[\nu[I_0(t_N) + \delta_I(\omega)\exp(i\omega t_N)]] \tag{28}$$

For this transformation to be stable $I \circ \nu$ needs be have a Jacobian with eigenvalues less than 1. The Jacobian of this transformation can be obtained by considering the Jacobian (linear response function) of the individual transformations. First, we consider the linear response of the transformation from current to firing rate:

$$\nu[I_0(t_N) + \delta_I(\lambda)\exp(\lambda t_N)] = \nu_0(t_N) + \delta_\nu(\lambda)\exp(\lambda t_N) \tag{29}$$

This can be obtained by calculating the dynamical change in mean voltage $f(t)$ and the resulting firing rate:

$$\nu(t) = \frac{1}{2\pi\sigma_V\sigma_{\dot{V}}}\int_{-\dot{f}(t)}^{\infty} d\dot{V}(t)(\dot{V}(t) + \dot{f}(t))\exp\left(-\frac{1}{2}\left(\frac{(V_{th} - V_0 - f(t))^2}{\sigma_V^2} + \frac{(\dot{V}(t))^2}{\sigma_{\dot{V}}^2}\right)\right). \tag{30}$$

Solving this integral we obtain:

$$\nu(t) = \frac{\exp\left(-\frac{(V_{th}-V_0-f(t))^2}{2\sigma_V^2}\right)\cdot\frac{\sigma_{\dot{V}}}{2}\left(\sqrt{2\pi}\mathrm{Erf}\left(\frac{\dot{f}(t)}{\sqrt{2}\sigma_{\dot{V}}}\right)\dot{f}(t) + \sqrt{2\pi}\dot{f}(t) + 2\sigma_{\dot{V}}\exp\left(-\frac{\dot{f}(t)^2}{2\sigma_{\dot{V}}^2}\right)\right)}{2\pi\sigma_V\sigma_{\dot{V}}}. \tag{31}$$

The linear response (expansion in the small parameter $f(t)$) is then given by:

$$\nu(t) = \nu_0 + \exp\left(-\frac{(V_0 - V_{th})^2}{2\sigma_V^2}\right)(\sqrt{2\pi}\sigma_V^2 f'(t) + 2\sigma_{\dot{V}}(V_{th} - V_0)f(t))\frac{1}{4\pi\sigma_V^3} \tag{32}$$

Now we address the mean voltage change $f(t)$ and its time derivative. We assume that the input is given by $X_0 + \delta_X\exp(i\omega t)$ and the resulting modulation of voltage $V_0 + f(t) = V_0 + f(\omega)\exp(i\omega t)$:

$$\tau_V f(\omega)i\omega\exp(i\omega t) = -(V_0 + f(\omega)\exp(i\omega t)) + \gamma_c(V_0 + f(\omega)\exp(i\omega t)) \tag{33}$$
$$+ \alpha(W_m + w(\omega)\exp(i\omega t)) + X_0 + \delta_X\exp(i\omega t)$$

$$\tau_w i\omega w(\omega)\exp(i\omega t) = -(W_m + w(\omega)\exp(i\omega t)) + \beta(V_0 + f(\omega)\exp(i\omega t)) \tag{34}$$

we obtain for $f(t)$ in leading order:

$$f(t) = \frac{\delta_X(1+i\tau_w\omega)}{-\alpha\beta + ((1-\gamma_c)+i\tau_V\omega)(1+i\tau_w\omega)} \tag{35}$$

$$w(t) = \frac{\beta\delta_X \exp(i\omega t)}{-\alpha\beta + ((1-\gamma_c)+i\tau_V\omega)(1+i\tau_w\omega)} \tag{36}$$

We now can write:

$$\nu(t) = \nu_0 + \frac{\delta_X \exp(-\frac{(V_0-V_{th})^2}{2\sigma_V^2} + it\omega)(-2\sigma_{\dot{V}}(V_{th}-V_0) - i\sqrt{2\pi}\sigma_V^2\omega)(1+i\tau_w\omega)}{4\pi\sigma_V^3(\alpha\beta - ((1-\gamma_c)+i\tau_V\omega)(1+i\tau_w\omega))} \tag{37}$$

Replacing $\lambda = i\omega$ we obtain the Jacobian of this transformation for excitatory neurons:

$$R_E(\lambda) = \frac{\exp(-\frac{(V_0-V_{th})^2}{2\sigma_V^2})(-2\sigma_{\dot{V}}(V_{th}-V_0) - \lambda\sqrt{2\pi}\sigma_V^2)(1+\tau_w\lambda)}{4\pi\sigma_V^3(\alpha\beta - (1+\tau_V\lambda)(1+\tau_w\lambda))} \tag{38}$$

A maximum of this response function $R_E(\omega)$ is located at $\omega_R$.

$$\omega_{RE} = \sqrt{\frac{\tau_w^2\left(2\tau_V^2C - \pi A\sigma_V^4\right) - \sqrt{B\tau_w^3(B\tau_w - 2T)\left(\pi^2A\sigma_V^8 - 2\pi\tau_w^4C\left(2(B-1)\tau_V\tau_w + T^2\right) + 4\tau_w^2\tau_V^2C^2\right)}}{\tau_w^3\left(\pi\sigma_V^4(2B\tau_V + \tau_w) - 2\tau_V^2\tau_wC\right)}} \tag{39}$$

Where $T = \tau_V + \tau_w$, $A = (\alpha\beta - 1)^2$, $C = \sigma_{\dot{V}}^2(V_0 - V_{th})^2, B = \alpha\beta$. This corresponds to a resonance peak whenever the right hand side of Eq. 39 is real. For inhibitory neurons that are connected through gap-junctions the corresponding results are:

$$R_I(\lambda) = \frac{\exp(-\frac{(V_0-V_{th})^2}{2\sigma_V^2})(-2\sigma_{\dot{V}}(V_{th}-V_0) - \lambda\sqrt{2\pi}\sigma_V^2)(1+\tau_w\lambda)}{4\pi\sigma_V^3(\alpha\beta - (1-\gamma_c+\tau_V\lambda)(1+\tau_w\lambda))} \tag{40}$$

The response of electrically unconnected inhibitory neurons can be obtained by setting $\gamma_c = 0$ (Eq. 3 in the main text). A maximum of this response function $R_I(\omega)$ displays is at $\omega_{RI}$

$$\omega_{RI} = \sqrt{\frac{\tau_w^2\left(2\tau_V^2C - \pi A\sigma_V^4\right) - \sqrt{B\tau_w^3((B+2\gamma_c)\tau_w - 2T)\left(\pi^2A\sigma_V^8 - 2C\pi\sigma_V^4K + 4\tau_w^2\tau_V^2C^2\right)}}{\tau_w^3(2B\pi\sigma_V^4\tau_V + ((\gamma_c-1)^2\pi\sigma_V^4 - 2C\tau_V^2)\tau_w)}} \tag{41}$$

Where $T = \tau_V + \tau_w$, $A = (\gamma_c + \alpha\beta - 1)^2$, $C = \sigma_{\dot{V}}^2(V_0 - V_{th})^2, B = \alpha\beta$, $K = (\tau_V^2 + 2B\tau_V\tau_w + (\gamma_c - 1)^2\tau_w^2)$. This corresponds to a resonance peak whenever the right hand side of Eq. 39 is real.

Next, we address the second transformation from firing rate change $\nu_E(t)$ to current. The mean current in Eq. 5 is

$$\begin{aligned} \dot{I}(t) &= \gamma_c[-(I(t) - \Gamma_{iI}\nu_I - \Gamma_{iE}\nu_E(t) - \mu_{ext})/\gamma_c + \alpha w(t) + I(t)]/\tau_V + \Gamma_{iE}\dot{\nu}_E(t) \\ \tau_w\dot{w}(t) &= -w(t) + \beta(I(t) - \Gamma_{iI}\nu_I - \Gamma_{iE}\nu_E(t) - \mu_{ext})/\gamma_c \end{aligned} \tag{42}$$

The last two equations need to be solved to obtain dynamical current response function. In the Fourier domain they read:

$$I(\omega) = \frac{\Gamma_{iE}(\alpha\beta - (1+i\omega\tau_V)(1+i\omega\tau_w))\nu_E(\omega)}{\alpha\beta + ((\gamma_c-1) - i\omega\tau_V)(1+i\omega\tau_w)}, \tag{43}$$

$$w(\omega) = \frac{\beta\gamma_c\Gamma_{iE}\nu_E(\omega)}{\gamma_c(\alpha\beta + (i(-1+\gamma_c)+\omega\tau_V)(-i+\omega\tau_w))} \tag{44}$$

Replacing $\lambda = i\omega$ we obtain the Jacobian of this transformation for gap-junction connected inhibitory neurons:

$$S_I(\lambda) = \frac{\tau_V(\alpha\beta - (1+\lambda\tau_V)(1+\lambda\tau_w))}{\alpha\beta - (1-\gamma_c+\lambda\tau_V)(1+\lambda\tau_w)} \tag{45}$$

For excitatory neurons, we obtain an analogous result

$$S_E(\lambda) = \frac{\tau_V(\alpha\beta - (1+\lambda\tau_V)(1+\lambda\tau_w))}{\alpha\beta - (1-\lambda\tau_V)(1+\lambda\tau_w)} \tag{46}$$

**Stability**

For the two dimensional $E-I$ model the following considerations lead to a stability condition:

$$\begin{pmatrix} \delta I_E \\ \delta I_I \end{pmatrix} = \begin{pmatrix} \Gamma_{EE}R_E(\omega)S_E(\omega) & \Gamma_{EI}R_I(\omega)S_I(\omega) \\ \Gamma_{IE}R_E(\omega)S_E(\omega) & \Gamma_{II}R_I(\omega)S_I(\omega) \end{pmatrix} \begin{pmatrix} \delta I_E \\ \delta I_I \end{pmatrix} \tag{47}$$

$$\det \begin{pmatrix} \Gamma_{EE}R_E(\omega)S_E(\omega) - 1 & \Gamma_{EI}R_I(\omega)S_I(\omega) \\ \Gamma_{IE}R_E(\omega)S_E(\omega) & \Gamma_{II}R_I(\omega)S_I(\omega) - 1 \end{pmatrix} \tag{48}$$

$$= (\Gamma_{EE}R_E(\omega)S_E(\omega) - 1)(\Gamma_{II}R_I(\omega)S_I(\omega) - 1) = \Gamma_{EI}R_I(\omega)S_I(\omega)\Gamma_{IE}R_E(\omega)S_E(\omega) \tag{49}$$

$$= (\Gamma_{EE}R_E(\omega)S_E(\omega) - 1)(|\Gamma_{II}|R_I(\omega)S_I(\omega) + 1) = |\Gamma_{EI}|R_I(\omega)S_I(\omega)\Gamma_{IE}R_E(\omega)S_E(\omega) \tag{50}$$

For networks with no recurrent connections ($\Gamma_{EE} = \Gamma_{II} = 0$) this condition can be simplified to:

$$-1 = |\Gamma_{EI}|R_I(\omega)S_I(\omega)\Gamma_{IE}R_E(\omega)S_E(\omega) \tag{51}$$

In this case the phase shifts from $R_I(\omega)$,$S_I(\omega)$, $R_E(\omega)$, and $S_E(\omega)$ should sum up to $\pi$ and the amplitude should fulfill $abs(|\Gamma_{EI}|R_I(\omega)S_I(\omega)\Gamma_{IE}R_E(\omega)S_E(\omega)) = 1$. Also the previous case of purely inhibitory network emerges in the limit $\Gamma_{EE} = \Gamma_{EI} = \Gamma_{IE} = 0$ and we obtain as previously:

$$\Gamma_{II}R_I(\omega)S_I(\omega) = 1 \tag{52}$$
